# Prospective, comparative clinical pilot study of cold atmospheric plasma device in the treatment of atopic dermatitis

**DOI:** 10.1038/s41598-021-93941-y

**Published:** 2021-07-14

**Authors:** Young Jae Kim, Dong Jun Lim, Mi Young Lee, Woo Jin Lee, Sung Eun Chang, Chong Hyun Won

**Affiliations:** Department of Dermatology, Asan Medical Center, Ulsan University College of Medicine, 88, OLYMPIC-RO 43-GIL Songpa-gu, Seoul, 05505 South Korea

**Keywords:** Medical research, Energy science and technology

## Abstract

Cold atmospheric plasma generates free radicals through the ionization of air at room temperature. Its effect and safety profile as a treatment modality for atopic dermatitis lesions have not been evaluated prospectively enough. We aimed to investigate the effect and safety of cold atmospheric plasma in patients with atopic dermatitis with a prospective pilot study. Cold atmospheric plasma treatment or sham control treatment were applied respectively in randomly assigned and symmetric skin lesions. Three treatment sessions were performed at weeks 0, 1, and 2. Clinical severity indices were assessed at weeks 0, 1, 2, and 4 after treatment. Additionally, the microbial characteristics of the lesions before and after treatments were analyzed. We included 22 patients with mild to moderate atopic dermatitis presented with symmetric lesions. We found that cold atmospheric plasma can alleviate the clinical severity of atopic dermatitis. Modified atopic dermatitis antecubital severity and eczema area and severity index score were significantly decreased in the treated group. Furthermore, scoring of atopic dermatitis score and pruritic visual analog scales significantly improved. Microbiome analysis revealed significantly reduced proportion of *Staphylococcus aureus* in the treated group. Cold atmospheric plasma can significantly improve mild and moderate atopic dermatitis without safety issues.

## Introduction

Artificial plasma has been used in the tissue removal, cauterization, or sterilization of medical instruments, being a technology with potential to be applied in various medical fields^1^. In particular, non-thermal atmospheric plasma, also known as cold atmospheric plasma (CAP) was getting attention due to its possibility to directly contact with the human body. In this context, CAP allows heat-sensitive targeting applications at low temperatures, without any specific extra cooling. As above-mentioned, CAP serves different medical purposes as a disinfecting and bleaching through the generation of free radicals including the reactive oxygen species or nitrogen oxygen species in combination with transient electric fields or UV radiation^2–4^. Recent studies updated that CAP promotes the wound healing process by reducing inflammation and oxidative stress^5,6^. Its potential roles in promoting angiogenesis, inducing proliferation, tissue oxygenation and migration of keratinocytes and fibroblasts have been also documented^7–9^. Moreover, CAP is also effective for relieving skin inflammation in mice with atopic dermatitis^10,11^.

Atopic dermatitis is a common chronic inflammatory skin disease that affects 10–20% of children and 1–3% of adults^12^. Since the severity of each individual varies, a systematic, and diverse therapeutic approach is required according to the patients’ clinical manifestations. Nonetheless, its known that several therapies, such as topical agents or oral medications still have potential side effects, especially if they are used for a long term. In addition, even under an appropriate treatment, most atopic lesions are likely to worsen rather than completely disappear like an external flame that never goes out. Therefore, an alternatively sustainable treatment without patients’ burden is urged to manage the localized atopic lesions. In this context, CAP device may serve as a novel treatment option for these indications.

Until now, limited studies are available regarding the clinical evidence of CAP treatment in atopic dermatitis patients^13,14^. This pilot study investigated the efficacy and safety of CAP in atopic dermatitis patients.

## Methods

### Patients

This study was approved by the institutional review board of Asan Medical Center (2020-0811). A prospective clinical study was performed, from May 20, 2020, to August 10, 2020. All the patients signed the informed patient. This study included patients with an age above 19 and with a diagnosis of atopic dermatitis of mild to moderate severity with symmetric skin lesions. The severity of disease was defined as “*mild*” if their Investigator’s Global Assessment (IGA) score 2 and “*moderate*” if their IGA score 3 (Supplementary Table 1). Moreover, only patients who had atopic dermatitis lesions with total body surface area (BSA) < 30% were included. The exclusion criteria included any history of systemic treatment of atopic dermatitis, such as corticosteroids, antibiotics, immunosuppressive agents, or phototherapy used within the last 4 weeks prior to the study. Any history of topical agents, such as topical corticosteroids, immunosuppressants, or antibiotics within 2 weeks also excluded. Patients who had other severe medical problems or mental illness, and who were pregnant were excluded from this study. Patients were only allowed to take oral antihistamines (n = 3) for the relief of symptom and to apply emollients during the whole study period.

### Plasma device and treatment protocol

**Figure 1:**
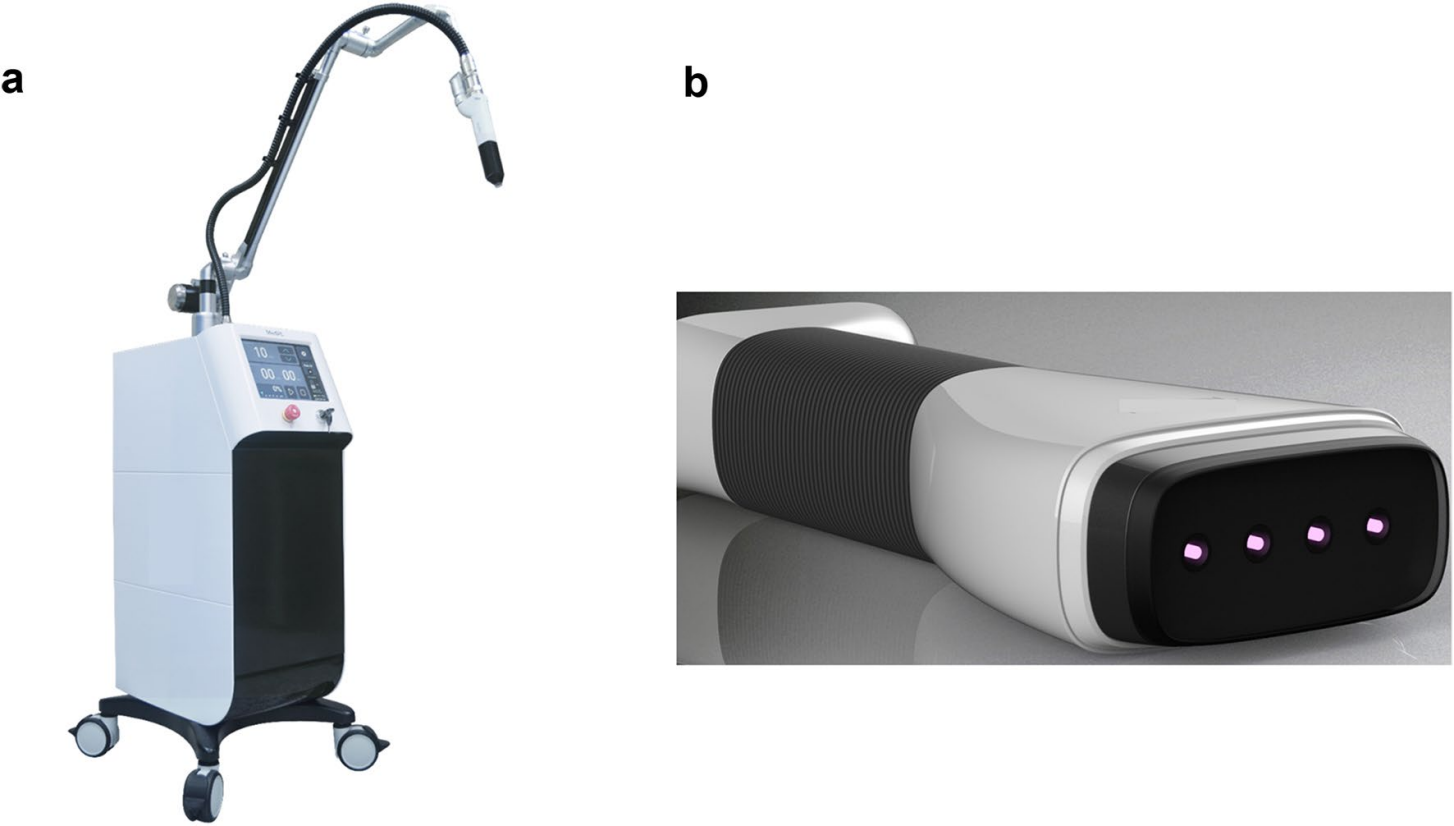
Cold atmospheric plasma (CAP) treatment device in this study, which uses argon gas to generate plasma. (**a)** Overall device appearance (**b**) A four-jet array handpiece was used in this study.

The device used during the study was a microwave-driven CAP device called MediPL Derm, developed by MediPL (Seoul, Korea) (Fig. 1a). This CAP device belongs to the indirect plasma type entrained with an argon gas flow^15,16^. A sham treatment device was also prepared, having the same appearance of the genuine plasma. CAP device employed the following setting to operate: microwave frequency 2.45 GHz ± 50 MHz, plasma power with 1.5 W at each electrode array, plasma temperature between the plasma device and target < 41 °C to prevent low temperature burn, continuous signal, four-array jet handpiece (Fig. 1b). Since the plasma intensity can vary depending on the flow rate, we maintained argon gas flow as 0.6 standard liter per minute (slm) per electrode to maintain a constant plasma generation. The change in the total ultraviolet index before and after using the plasma device was 0.0055. Supplementary Fig. 1 shows optical emission spectrum of plasma device in this study. Sham treatment device employed the same setting as the CAP device, except for the absence of microwave for ignition and for that reason no plasma energy generates.

For each patient, CAP or sham treatment was randomly assigned and used to treat the symmetric lesions. We defined symmetric lesions as the skin lesions existing on both sides of anatomical sites of extremities. For instance, if the patients with atopic dermatitis (AD) lesions on their both arms are randomly assigned to receive plasma treatment on the right arm, the patients will receive sham treatment on the left arm. Before the treatment, we wiped off the lesions with a normal saline soaked gauze. The plasma or sham treatment device was set to keep the distance between the lesion and device within 5 mm. The treatments were made during five minutes in each session.

Plasma and sham treatment were weekly used at week 0 (baseline), 1 and 2, being asked the patients to visit at week 4 for follow-up assessment.

### Assessment of clinical severity in atopic dermatitis

At week 0, 1, 2, and 4, all the patients of IGA were assessed with a modified ADAS (Atopic Dermatitis Antecubital Severity) score, EASI (Eczema Area and Severity Index) score, SCORAD (Scoring of AD) score, and pruritic visual analog scale (VAS) score. The modified ADAS (Atopic Dermatitis Antecubital Severity) score is calculated by multiplying the intensity of inflammation by the area of an antecubital eczematous lesion^17^. The intensity of inflammation is evaluated using a 6-grade scale (0, absence; 1, mild; 2, mild-moderate; 3, moderate; 4, severe; and 5, very severe) for erythema, scale and excoriation on skin lesion respectively. The visual analog scale (VAS) is a scale consisting of 100 mm long line and a single question. In pruritus VAS, we asked every patient the severity of subjective pruritus in last three days. Scale 0 means "never felt itching sense", otherwise scale 10 means "most severe itching sense". Then the patient was asked to mark on the scale as they experienced^18^. The improvement achieved was compared (the degree of improvement) between the treatment group and the control group after treatments. Transepidermal water loss (TEWL) was assessed in patients with AD lesions using tewameter and corneometer (Courage-Khazaka, Köln, Germany) before the treatment (week 0) and at week 4. The clinical effectiveness of CAP was evaluated by comparison with the CAP treated arm and the sham treated counterpart using IGA, modified ADAS score, and TEWL. In addition, overall clinical severity of each patient was also assessed before and after the treatment, using EASI score, SCORAD score, and VAS score.

### Skin microbiome study

We swiped the lesions with an aseptic cotton swab for a minute to acquire the sample for microbial analysis. We collected patients’ bacterial samples just before the first treatment session (week 0) and at week 4. Microbial analysis was done through 16s-rRNA gene sequencing.

### Statistics

Data were analyzed using SPSS 21.0. Paired t-test was used for continuous variables analysis, such as modified ADAS score, EASI score, SCORAD score, pruritus VAS score and *Staphylococcus aureus* (*S. aureus*) loading. Chi-square test was used for the analysis of categorical variables, namely proportion of IGA improvement.

## Result

Twenty-two (13 females and 9 males) Korean patients were enrolled in the present study. The patients included in this study completed three treatment sessions and follow-up visit. The mean patient age was 27.2 ± 6.9 years (range, 20–42 years). Among 22 patients, 9 patients revealed a mild AD and 13 patients were moderate AD (Table 1). The mean EASI score of mild AD patients was 2.11 ± 1.14. The mean EASI score of moderate AD patients was 5.72 ± 2.71.

**Table 1:** Characteristics of the patients.

**Patient no.**	**Sex/age**	**Baseline**				**Treatment site**
		**IGA (Lt/Rt)**	**Severity**	**EASI**	**Total serum IgE (kU/I)**	
1	F/29	3/3	Moderate	5.2	246	Upper arm
2	M/23	3/3	Moderate	5.6	3786	Popliteal area
3	M/41	3/3	Moderate	2.7	72.8	Shin
4	M/23	3/3	Moderate	7.5	1454	Antecubital area
5	F/39	2/2	Mild	1.6	25	Medial knee
6	F/22	3/3	Moderate	2.4	671	Antecubital area
7	F/23	3/3	Moderate	8.8	124	Popliteal and antecubital area
8	F/19	2/2	Mild	2.6	116	Popliteal and antecubital area
9	M/33	2/2	Mild	1.0	2684	Upper arm
10	F/22	3/3	Moderate	3.4	< 2	Antecubital area
11	F/25	2/2	Mild	0.8	54.3	Antecubital area
12	F/32	3/3	Moderate	7.6	3901	Antecubital area
13	F/22	3/3	Moderate	2.4	662	Antecubital area
14	M/20	3/3	Moderate	9.4	2714	Antecubital area
15	M/24	2/3	Mild/moderate	4.0	645	Antecubital area
16	M/28	3/3	Moderate	5.3	2241	Antecubital area
17	M/32	3/3	Moderate	10	1496	Antecubital area and elbow
18	F/42	2/2	Mild	4.6	> 5000	Popliteal and antecubital area
19	M/31	2/2	Mild	2.4	2737	Antecubital area
20	F/28	2/2	Mild	1.4	1116	Antecubital area
21	F/20	2/2	Mild	2.4	63.4	Shin
22	F/21	2/2	Mild	2.2	37.2	Popliteal area

**Figure 2:**
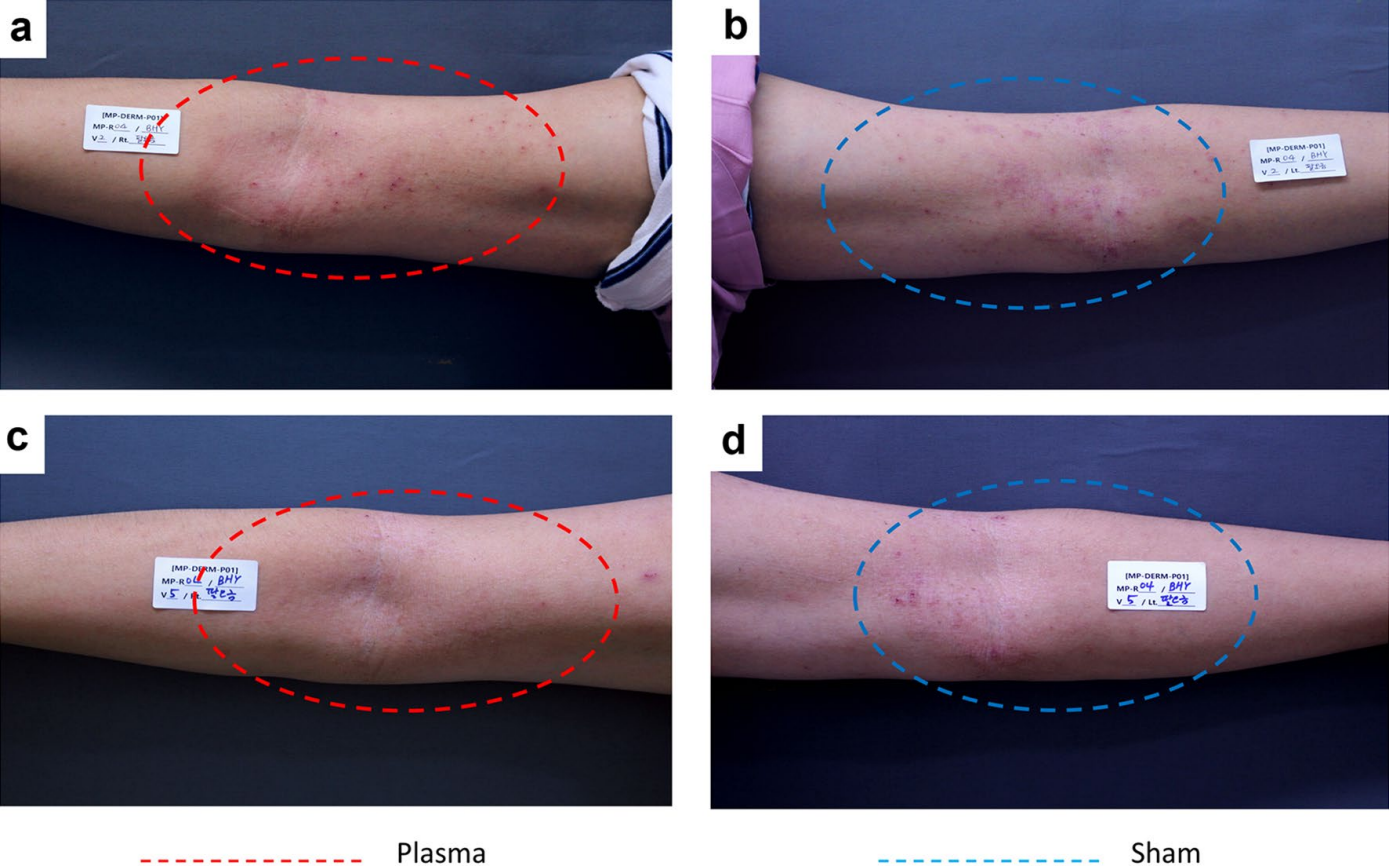
Three treatment sessions of cold atmospheric plasma improved the severity of atopic skin lesion. A 23-years old male with favorable results after CAP treatment compared to sham treated arm. Erythema and papules/indurations noticeably diminished. (**a**) plasma treated arm, at week 0 (**b**) sham treated arm, at week 0 (**c**) plasma treated arm, at week 4 (**d**) sham treated arm, at week 4.

**Figure 3:**
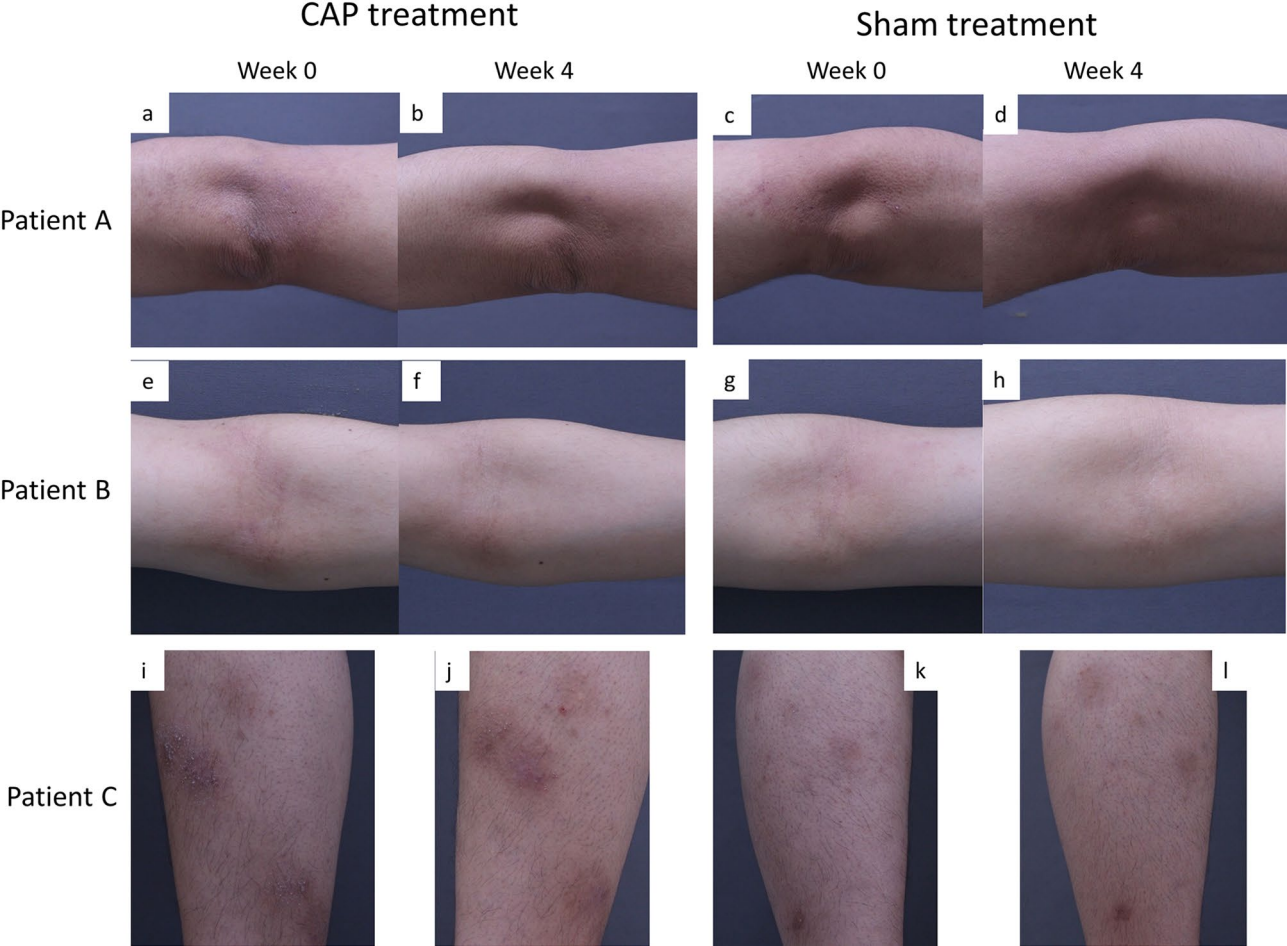
Clinical photography of representative subjects. (**a**–**d**) Patient A, 32-years old man with modified ADAS score 85 on his CAP treated arm at week 0. After three treatment sessions, modified ADAS score was improved to 0. This case particularly improved and almost all the skin lesions in treated arm have disappeared, whereas still mild erythema remained with pruritus yet on sham treated side. (**e**–**h**) Patient B, 28-years old man with modified ADAS score 18 on his CAP treated arm at week 0 and score 0 at week 4. (**i**–**l**) Patient C, 20-years old woman with modified ADAS score 21 on her CAP treated shin at week 0 and score 15 at week 4.

**Figure 4:**
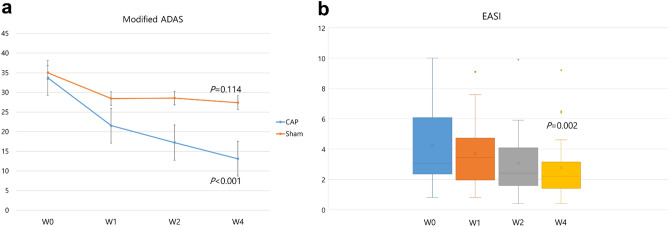
Clinical indexes along with follow-up period. (**a**) At week 4, modified ADAS score was significantly improved in plasma treated arm (*p* value < 0.001), whereas score was not significantly changed in sham treated arm (*p* value = 0.114). (**b**) Patients’ EASI score was significantly improved at week 4 (*p* value = 0.002).

Several clinical indexes showed a statistically significant improvement after CAP treatment compared to sham treatment (Figs. 2, 3). The mean modified ADAS (mean ± standard deviation) score at baseline was 33.73 ± 21.21 in CAP group and 35.02 ± 15.13 in sham group, respectively. Along with the study, the mean modified ADAS score in CAP group was decreased to 21.56 ± 16.07 at week 1 and 17.23 ± 18.54 at week 2. After three treatment sessions, at week 4, the mean modified ADAS score in CAP group was 13.12 ± 15.92. CAP treated atopic skin lesions showed a significant improvement in modified ADAS score at week 4 (*p* value < 0.001) compared to sham treated lesion, which showed no statistically significant improvement (*p* value = 0.114) (Table 2). The mean EASI score at baseline was 4.24 ± 2.82. This value gradually decreased to 3.70 ± 2.23 at week 1 and 3.05 ± 2.22 at week 2. At the end of the study, the mean EASI score was 2.76 ± 2.18, being significantly improved as compared to baseline (*p* value = 0.002) (Fig. 4). Another atopic severity score, SCORAD had also been significantly improved. The mean SCORAD score at baseline was 29.63 ± 9.42, which significantly decreased as much as 19.43 ± 11.50 at the end of study (*p* value = 0.001). Concerning the pruritic VAS, it was significantly improved at the end of study from 5.14 ± 1.97 at baseline to 3.83 ± 2.12 (*p* value = 0.032).

**Table 2:** Summary of clinical results.

	W0 (baseline)	W1	W2	W4
**Modified ADAS**				
CAP	33.73 ± 20.70	21.56 ± 15.70	17.23 ± 18.10	13.12 ± 15.60
*p* value				< 0.001*
Sham	35.02 ± 14.80	28.45 ± 15.00	28.57 ± 18.70	27.39 ± 23.20
*p*-value				= 0.114
EASI	4.24 ± 2.76	3.70 ± 2.18	3.05 ± 2.16	2.76 ± 2.13
*p* value				= 0.002
**Number of patients showing improved IGA score**				
CAP	N/A	6	7	15
Sham	N/A	3	4	9

*ADAS* atopic dermatitis antecubital severity, *CAP* cold atmospheric plasma, *EASI* eczema area and severity index, *IGA* investigator’s global assessment.

The proportion of lesions that showed an improved IGA score at week 4 was 68.2% (15/22) in CAP group and 40.9% (9/22) in sham group. Although without a statistically significant difference, there was a tendency to improve of IGA score after CAP treatments (*p* value = 0.069). The mean TEWL at plasma treated arms values were not significantly changed, from the baseline 24.40 ± 11.93 to 24.24 ± 7.51 g h^−1^ m^−2^ at week 4 (*p* value = 0.960). The decrease in the mean value of corneometer was not statistically significant as well, from 45.71 ± 13.47 at baseline to 40.57 ± 8.94 at 4 weeks (*p* value = 0.133). Notably, when the groups were divided according to total serum IgE levels, the group with total serum IgE > 100 kU/I showed a significant decrease in modified ADAS score (*p* value < 0.001) and EASI score (*p* value = 0.002) after the treatment of CAP at final follow-up assessment. However, the group with a total IgE ≤ 100 kU/I revealed no significant improvement in the EASI score at final follow-up assessment (*p* value = 0.730).

Concerning the microbial analysis, only nine patients completed the study due to the specimen adequacy and due to the refusal of some patients. We focused on the effect of CAP on the proportion of *S. aureus* over the whole microbiata on the lesion. The mean proportion of *S. aureus* at baseline was 23.03% in CAP group, being 16.26% in sham group. After the treatment sessions, the mean proportion of *S. aureus* was 10.14% in CAP group, while 15.29% in sham group. The difference in the reduction of *S. aureus* counting was statistically significant between CAP and sham group (*p* value = 0.047).

Side effects were monitored by the investigators (YJK, CHW) during every visit through the physical examination. During the treatment and the follow-up period, no severe side effects were observed, including burn, prolonged erythema, and pain related to CAP treatment. None expressed slight warmth on the treated lesion after the CAP treatments was reported.

## Discussion

AD occurs as a result of complex interactions, such as skin barrier dysfunction, immunologic dysfunction, environmental, and genetic factors^19^. The chronic course of AD manifested with dry and itchy skin presentation may severely deteriorate the patients’ quality of life. In particular, vicious cycles of the scratch and itch cycle cause a continuous mechanical damage to the skin, accelerating several inflammatory reactions, which contributes to a prolonged course of the disease^20^. Therefore, stopping the repetitive scratch and itch cycles and restoring the damaged skin barrier may be an effective therapeutic strategy for itchy atopic skin. Notably, it has been reported that the symptoms of pruritic skin improved significantly with CAP application for an average of 4.7 times a day for 2 min^14^. Moreover, CAP can promote the skin regeneration and wound healing by increasing the interleukin(IL)-6 and transforming growth factor-beta (TGF-b) in the skin^21,22^. Moreover, stimulated angiogenesis during wound healing process was also noted by upregulating growth factors, such as vascular endothelial growth factor, epithelial growth factor, fibroblast growth factor, or by production of reactive oxygen species^23^. In the atopic mice skin, it was observed that the treatment of keratinocytes with CAP effectively downregulated the expression of CCL11, CCL13, and CCL17, which are main driving chemokines for the induction of AD^24^. In this human clinical study, patients with AD showed significant improvement in pruritic VAS (*p* value = 0.032) and atopic severity scores, including modified ADAS (p < 0.001), EASI (p = 0.002), and SCORAD score (*p* value = 0.001). These results support that CAP would be helpful to relieve symptoms of AD by reducing chronic itching and promoting the recovery of secondary wounds caused by scratching.

Interestingly, CAP also revealed antibacterial effects in the skin. Isbary et al. reported that the bacteria in the wound area were reduced when an average of 7.86 treatments with CAP were performed, daily for 5 min for chronically unhealed wounds^25^. The bacteriocidal effect of CAP is meaningful in the treatment of AD, because the skin microbiome and its fluctuations are directly associated with AD^26^. Up to 90% of atopic patients showed dominant *S. aureus* colonization in skin microbiome and this increased colonization was known to be prominent in disease flare-up period^27,28^. Recent investigations in children suggested that cutaneous *S. aureus* colonization may lead to disease development, as well as, function as an exacerbating factor of AD^29^. Therefore, therapeutic attempts to restore the natural skin microflora by reducing colonized *S. aureus* are plausible strategies to improve the treatment of AD^30,31^. In this context, medications for AD, such as topical corticosteroids and topical calcineurin inhibitors, play a role in restoring the diversity of skin microbiome^32^. In our microbiome analysis, the mean proportion of *S. aureus* was significantly decreased in CAP treated group comparative to sham treated group (*p* value = 0.047). Considering that atopic patients are susceptible to secondary bacterial infection due to a damaged skin barrier, the results of the present study are noteworthy. Indeed, CAP can improve atopic symptoms by promoting the recovery of the diversity of skin microbiome, as well as, to prevent secondary infection of the skin.

We attempted to demonstrate the therapeutic effect of CAP by measuring the change in TEWL as in the previous study^33^. However, the average decrease in TEWL was not significant before and after the treatment of CAP. The function of the skin barrier is affected by multiple extrinsic factors, such as temperature, humidity, and ultraviolet irradiation^34,35^. Recent studies have also revealed that the function of the skin barrier is associated with patients’ stress^36^. Taken together these findings, there are some limitations in understanding the impact of CAP on the skin barrier function with changes of TEWL.

It is remarkable that there is a difference in response to CAP treatment according to total serum IgE. The group with total IgE > 100 kU/I was significantly improved in modified ADAS score (*p* value < 0.001) and EASI score (*p* value = 0.002) after CAP treatments. These results are due to the difference between the extrinsic and intrinsic types, suggested subtypes of ADs. Extrinsic and intrinsic types are defined according to IgE-mediated sensitization, according to the presence of specific IgE for food and environmental allergens^37^. Since total serum IgE levels are significantly correlated with the allergen-specific IgE status^38^, total IgE is considered as a clinical marker to differentiate between the extrinsic and intrinsic types in both adults and children^38,39^. Thus, the group with total IgE > 100 kU/I are considered as extrinsic type of AD which has been described with elevated serum total IgE and disrupted skin barrier function^37,40^. Ricci et al. also found that the extrinsic type AD showed a higher colonization of *S. aureus* compared to the intrinsic type children (71% vs. 49%)^41^. CAP treatment appears to improve the AD by restoring damaged skin barrier with antibacterial effect especially for causative *S. aureus* considering that CAP treatment is particularly effective in the group with elevated total IgE in this study, as above-mentioned.

The present pilot study has several limitations. First, this study was a single-center study with a small sample size. Further studies with multi-centered, large sample size are warranted. Second, patients with severe AD had not been included, because most of them were relied on oral immunosuppressants and reluctant to attempt CAP treatment alone. To evaluate the therapeutic effect of CAP alone, we considered that it is desirable to evaluate patients with mild to moderate AD in first place. Third, potential bias may have occurred due to the limited data on the microbiome analysis assessed in this study. Fourth, there is a possibility of “abscopal effect” and immune-triggering effect after the CAP treatment^42^. Nevertheless, our study is still noteworthy, because this is the first prospective clinical trial of CAP device on AD patients. Until now, there have been no clinical trials of CAP in human AD. In addition, CAP treatment is easy to apply in that it does not have safety problems at all, unlike oral immunosuppressant which have side effects in long term use. This strong point implies that CAP treatment also showed the potential as a burden-free adjuvant therapy. In fact, most of patients in this study showed a substantial satisfaction with CAP treatment without any discomfort. Combinatory treatment with topical agents would be effective treatment options, because CAP is also known to enhance cutaneous transdermal drug delivery by regulating E-cadherin-mediated cell junction^43^. Choi et al. demonstrated that combinatory treatment of CAP with conventional anti-inflammatory topical agents could be effective for shortening the healing duration and minimizing the drug amount^24^. In this study, the authors showed that the application of CAP or 1% hydrocortisone alone did not reduce the DNCB-mediated epidermal thickening in mice, but the thickened epidermis was restored by the combined treatment. Further clinical investigations with real human atopic skin are required to prove the effectiveness of CAP as an adjuvant treatment.

In conclusion, CAP has the potential to effectively improve the severity of mild and moderate AD by recovering the diversity of skin microbiome, as well as, promoting wound healing for damaged skin barrier. Patients’ subjective pruritic symptoms will also be alleviated by the treatment of CAP without safety issues. This study supports new findings in treatment response data of CAP would update the new therapeutic approach in the AD field.

## Supplementary Information

Supplementary Figure 1.

Supplementary Table 1.
