# Supplementary material for: Prospective, comparative clinical pilot study of cold atmospheric plasma device in the treatment of atopic dermatitis
Source: Sci Rep. 2021 Jul 14;11:14461. doi: 10.1038/s41598-021-93941-y (PMC8280139; doi:10.1038/s41598-021-93941-y)
Supplement: Supplementary file 1 — Supplementary Figure 1. [file 41598_2021_93941_MOESM1_ESM.docx]

**Supplementary Figure 1. Optical emission spectrum of cold atmospheric plasma device in this study.**
