# Supplementary material for: Prospective, comparative clinical pilot study of cold atmospheric plasma device in the treatment of atopic dermatitis
Source: Sci Rep. 2021 Jul 14;11:14461. doi: 10.1038/s41598-021-93941-y (PMC8280139; doi:10.1038/s41598-021-93941-y)
Supplement: Supplementary file 2 — Supplementary Table 1. [file 41598_2021_93941_MOESM2_ESM.docx]

**Supplementary Table 1. Investigator’s Global Assessment (IGA) score**

| **Score** | **Grade** | **Description** |
| --- | --- | --- |
| 0 | Clear | No inflammatory signs of atopic dermatitis |
| 1 | Almost clear | Just perceptible erythema, and just perceptible papulation/infiltration |
| 2 | Mild | Mild erythema, and mild papulation/infiltration |
| 3 | Moderate | Moderate erythema, and moderate papulation/infiltration |
| 4 | Severe | Severe erythema, and severe papulation/infiltration |
| 5 | Very Severe | Severe erythema, and severe papulation/infiltration with oozing/crusting |
